# Public management in turbulent times: COVID‐19 as an ecosystem disruptor

**DOI:** 10.1111/1467-8500.12525

**Published:** 2021-11-22

**Authors:** Erik Eriksson, Christian Gadolin, Göran Lindahl, Patrik Alexandersson, Johanna Eriksson

**Affiliations:** ^1^ Division of Service Management and Logistics, Department of Technology Management and Economics, Centre for Healthcare Improvement Chalmers University of Technology Gothenburg Sweden; ^2^ Department of Work Life and Social Welfare University of Borås Borås Sweden; ^3^ Department of Health Sciences University West Trollhättan Sweden; ^4^ Division of Building Design, Department of Architecture and Civil Engineering, Centre for Healthcare Architecture Chalmers University of Technology Gothenburg Sweden

**Keywords:** COVID‐19, ecosystem, healthcare, public service logic, resource integration

## Abstract

The decentralisation of Swedish healthcare closer to citizens has been slow. Drawing from empirical material of the reform prior and amidst the COVID‐19 pandemic, this paper argues that the pandemic has disrupted the healthcare ecosystem. Consequently, citizen‐centred collaborations have accelerated integration of resources (such as knowledge and skills) across organisational, hierarchical and professional borders. However, collaborations have been delimited to traditional healthcare providers, neglecting the resources of citizens and other actors to be used to improve service delivery. The pandemic has revealed strengths and weaknesses with the prevailing healthcare ecosystem that post‐COVID‐19 public management must address, both theoretically and practically. Theoretically, the paper contributes to the development of a public service logic, addressing both strengths and difficulties with the logic in turbulent times. Practically, the empirical descriptions contribute to improved understanding of public service delivery reform and how it is impacted during the pandemic.

## Abstract 2

Turbulent times, such as the COVID‐19 pandemic, may accelerate reforms in a desired direction. However, expected enablers for value co‐creation in public service ecosystems, such as information technology, may be underused, despite the sense of urgency.

## 1 INTRODUCTION

The COVID‐19 pandemic echoes the risks typical of, and caused by, late modernity (e.g., Bauman, 2000), threatening humanity by moving beyond space (global impact) and time (inter‐generational repercussion). Previously, the consequences of COVID‐19 reached far beyond infections and deaths to people losing their businesses and employment, reinforcement of social problems and exclusion (e.g., Martin‐Howard & Farmby, 2020; Rauhaus et al., 2020). Although many countries have tried to counteract the virus through regulations and lockdowns, Sweden chose an alternative – and, some have argued, controversial – approach (particularly in the first wave, during spring 2020), largely keeping daycares, schools, and industries open and building on the voluntariness of citizens to follow recommendations from responsible public agencies and medical expertise (Pierre, 2020). However, COVID‐19 mortality rates have been substantially higher in Sweden than they have in the other Scandinavian countries (Christensen & Lægreid, 2020). An interim report from the Swedish Corona Commission (SOU, 2020a) focusing on elderly care concluded that regulations, accountability, and medical competence were lacking in Swedish elderly care and that this was why 90% of those who had died from COVID‐19 were at least 70 years old. Half of those lived at special accommodation facilities for the elderly.

Similar to Bauman (2000), public administration and management (PAM) researchers have argued that the challenges characterised in contemporary society are more complex, or ‘wicked’, than before (e.g., Bryson et al., 2017). Not only are poverty, climate change, and pandemics unruly and hard to address (Christensen, 2012; Geuijen et al., 2017), they are also hard to define because of the lack of standard solutions, unclear causalities, and goal conflicts (Peters & Pierre, 2017; Pollitt & Bouckaert, 2017). Because they are societal and global challenges (Bryson et al., 2017; Crosby et al., 2017), it is likely that the responsible public organisation will not be able to deal with this type of challenges alone. Unfortunately, traditional public administration and approaches from manufacturing industry have created inward‐oriented public organisations focusing on internal processes, standardisation, and rules (Grönroos, 2019; Osborne, 2020), fit to address simple challenges only (Ansell et al., 2020). Instead, varieties of governance approaches (e.g., Ansell & Gash, 2008; Dunleavy et al., 2006; Klijn & Kopenjaan, 2012) are argued to have a better fit in times of complexity, particularly by recognizing the full plurality of actors in, and across, societies in public service delivery (Bryson et al., 2017; Mintzberg, 2015; Sørensen & Torfing, 2011).

In various publications, Ansell and colleagues (e.g., Ansell et al., 2020; Ansell & Trondal, 2018) have argued that the simple–complex aspect of challenges is not sufficient. Not least, the COVID‐19 pandemic has recognised the aspect of turbulence (Ansell et al., 2020). Turbulent problems are not only complex, but also unpredictable, uncertain, inconsistent, and surprising (Ansell & Trondal, 2018). However, these characteristics are also commonly mentioned as typical for complex problems as well (e.g., Christensen, 2012; Geuijen et al., 2017). Nevertheless, complex challenges typically assume a relatively stable context for addressing these challenges and therefore make it relatively easy for public organisations to mobilize relevant actors in governance processes (Ansell et al., 2020). Instead, ‘the notion of turbulence aims to capture the increasingly volatile *context* for complex problem‐solving’ (Ansell et al., 2020, 3, our emphasis).

The turbulence of problems embedded in equally turbulent contexts must be taken into consideration, calling for public innovation, cross‐boundary collaborations, and strategies supporting and facilitating flexible and adaptive adjustments taking emerging opportunities and options into consideration. Here, we believe that the public service logic in general (e.g., Osborne, 2020), and the ecosystems approach within it (e.g., Petrescu, 2019), has much to offer by recognising the contribution of a multiplicity of actors across sectors in society. Conversely, the atypical situation of the pandemic may also contribute to the development of public service logic. Drawing on empirical material in the Swedish healthcare system, both pre‐COVID‐19 and amidst the pandemic, this paper theorizes how COVID‐19 may disrupt the healthcare ecosystem, which may cause, and accelerate, both desirable change within the ecosystem as well as exposing challenges caused by predominant ways of managing public services. The research question addressed is: *How does healthcare actors’ integration of resources enable and hinder decentralisation of healthcare closer to people during non‐turbulent times (pre‐COVID‐19) and during turbulent times (during COVID‐19)?*

In this paper, by comparing the effects of resource integration before and during the COVID‐19 pandemic, we seek to contribute to the development of a public service logic by elaborating on how ecosystems may respond to challenges in turbulent times. A central feature of the ecosystems literature is how a variety of actors are basically doing the same thing: exchanging and integrating resources (especially knowledge, skills, experience, etc., but also material resources) to create value for themselves, others, and society (Osborne, 2020; Vargo & Akaka, 2012). Therefore, in this paper we focus on how these resources are integrated (or not) during the COVID‐19 pandemic. The public service ecosystem literature is only just evolving, and empirical cases are scarce.

The remainder of this paper is organised as follows. Next, the emerging public service logic is presented, with extra focus on service ecosystems and how resources may be misused or neglected to be used. The methods section accounts for the Swedish healthcare system, the reform in question, and interviews and focus groups as primary methods for data collection. The findings are then presented chronologically: before and amidst the COVID‐19 pandemic. Then, by comparing to the pre‐COVID state, we discuss how the ongoing pandemic is disrupting, or unlocking, established practices and relations in the healthcare ecosystem in ‘desired’ ways, at the same time exposing well‐known challenges. The paper concludes by presenting relevance and contribution to theory and practice/policy.

## 2 PUBLIC SERVICE LOGIC

In recent decades, the PAM literature has drawn from a goods‐manufacturing logic, which has entailed a linearity by focusing on standardised processes and inwardness by focusing on improving internal efficiency (Grönroos, 2019; Osborne, 2020). These foci have created a notion of value to be created within public service organisations and then delivered to relatively passive service users as ‘value‐in‐exchange’ (Engen et al., 2021). Moreover, it has also been argued that these inward‐oriented public organisations are often unfit to address the more complex challenges since it requires a great deal of collaboration, both among organisations and with citizens as well as service users (Eriksson et al., 2020).

The intangibility feature of services (e.g., Parasuraman et al., 1985) entails that public service delivery and consumption cannot be separated, which is one reason why public service logic is less occupied with internal production processes and more with the provider–user interaction – also referred to as ‘moments of truth’ (Normann, 2001) – in which both parties participate in the production of the service equally as co‐producers (Hardyman et al., 2015). In a public service logic, value is primarily understood to be realised by the user during usage as ‘value‐in‐use’; therefore, the provider can offer potential value only, as value propositions: ‘more or less standardised configurations of resources’ (Skålén et al., 2018, p. 702). The user may contribute to develop services with the provider as a co‐producer to benefit not mainly itself, but others as well (Eriksson, 2019).

### 2.1 Public service ecosystems and resource integration

Generic service logic has increasingly focused on value creation beyond the provider‐customer dyad to include a multiplicity of actors contributing to resource integration (Akaka et al., 2013; Vargo & Lusch, 2016). These service ecosystems are defined as ‘relatively self‐contained, self‐adjusting systems of resource‐integrating actors connected by shared institutional logics and mutual value creation through service exchange’ (Vargo & Akaka, 2012, 207). Lately, public service logic has also expanded beyond user focus to emphasize the contributions of resources from a multiplicity of actors: public, private, third sectors, and service users (Eriksson & Hellström, 2021; Osborne et al., 2015). Trust and relationships are essential in this view as enablers for the integration of resources (Eriksson et al., 2020). In an ecosystems view, resource integration is argued to be mutually beneficial for all involved actors (Kinder et al., 2020; Petrescu, 2019) and to lead to value at the different levels of individual user, groups, and the societal level (Cluely et al., 2020; Dudau et al., 2019). In this sense, public services should be understood not only as the concern for the responsible public service organisation, but as a system (Osborne, 2020; Radnor et al., 2014), and consequently, managing the relationships among the involved system‐actors becomes essential (Osborne et al., 2015).

In a public service logic, to understand these systems, one must understand how they come to use during usage by the involved actors, since value cannot be delivered as embedded in policies, resources, and so forth (Osborne, 2020). However, empirical cases elaborating on public service logic remain scarce, not least addressing components of public service ecosystems, such as the multiplicity of actors (often across sectors) and their expectations and experiences of the service; the societal norms, beliefs, and values as prerequisites in such system; and the resources (knowledge and skills, infrastructure such as technology, as well as the spatial locus of delivered service) the actors bring to the system (Akaka et al., 2013; Eriksson, 2019; Osborne, 2020; Vargo & Akaka, 2012). The present paper focuses on the latter.

Like New Public Management, the public service logic draws from the developments in the private sector, and differences between the sectors are, with a few exceptions (e.g., Grönroos, 2019), well established as an important aspect of the theoretical developments (Osborne, 2020). One such difference is the notion of values; rather than value for the individual ‘customer’, public services also need to address public values (Alford, 2016), such as the common good, public interest, and democratic ideals (Beck Jørgensen & Bozeman, 2007). The ecosystems approach is particularly important for addressing public values on the society level since these cannot be analyzed at the individual level alone and must instead be understood in the value co‐creation process occurring among actors across a multiplicity of levels (Petrescu, 2019).

Despite the centrality of resource integration in the ecosystems view and the premise that they contribute to the creation of value (Vargo & Akaka, 2012; Vargo & Lusch, 2016), resources may also be used – or fail to be used at all – in an unwanted or unexpected way (Plé & Cáceres, 2010), leading to resource misuse and the destruction of value, which addresses the call for a more balanced notion of value creation ‘in which positive and negative coexist’ (Cluley et al., 2020, p. 1). The misuse, or failure to use, may be accidental or unintentional as well as deliberate and intentional (Plé & Cáceres, 2010) and may lead to lost relationship and benefits caused by trust (Järvi et al., 2018). Regarding value creation between organisational collaborations, Chowdhury et al. (2016) found opportunism, role conflicts and ambiguity, misunderstandings, and power plays to mobilize resources could lead to value destruction for the actors in the ecosystem.

## 3 METHODS

### 3.1 Setting: Care closer to citizens

The Swedish healthcare system is decentralised: the national level is responsible for legislating and establishing guidelines and principles; the 21 regions are responsible for offering care at the primary care or hospital level; and since a reform in 1992, 290 municipalities have been responsible for offering care at special accommodations or patients’ homes, particularly the older population (SOU, 2016). Clinical results, based on defined diagnostics rather than patient‐holistic aspects, are relatively good in the Swedish healthcare system, which scores worse than comparable countries in regard to patient‐centeredness (Vårdanalys, 2014) and collaboration among healthcare providers (SOU, 2016), which has caused coordination problems between providers and created a fragmented system that is difficult for patients to navigate (Eriksson et al., 2020).

A few years before the COVID‐19 pandemic, a reform gradually emerged to move healthcare closer to the citizens in Swedish healthcare and elderly care. Moving healthcare physically closer to the patient would increase efficiency, accessibility, equality, and patient‐centeredness, often by providing services at primary care or patients homes rather than healthcare facilities that are remote for the patients (SOU, 2020b). While specialised competence at the hospitals is still required, it is supposed to support and collaborate with healthcare providers at lower levels of care. This move has a substantial impact on facilities and spatial aspects, as well as patient‐staff role casting and interaction, not least when patients’ homes and citizens’ public spheres become healthcare facilities enabled by digital tools and new ways of communicating (SOU, 2020b).

### 3.2 Data collection and analysis

In this paper, aspects addressing care closer to patients were collected in two different time phases: prior and amidst the COVID‐19 pandemic. Both cases focus on resource integration relevant for the respective cases at the time: the integration of mainly intangible resources (knowledge, skills, experiences, etc.) possessed by various actors in the ecosystem that were integrated (or should be integrated, but were not) in order to contribute to mutual value creation for the involved actors as well as the societal level.

The interviews during the pandemic were conducted during June and August of 2020, a time when most people thought that the pandemic had already peaked, and few considered the possibility of a second wave (which became a reality during the autumn of 2020). Table 1 provides an overview of data collection.

**TABLE 1:** Data collection

Phase	**Data collection method**	**Number of participants**	**Sex**
Pre‐COVID‐19 (2017−2018)	Focus groups (2)	11 (7 and 4)	Eight female, three male
	Individual interviews	6	Four female, two male
During COVID‐19 (2020)	Individual interviews	29	22 female, seven men
Total		46	34 female, 12 male

In the pre‐COVID‐19 phase, data were collected through six semi‐structured individual interviews and two semi‐structured focus group discussions (Morgan, 1996). Participants in both individual and focus group interviews were either members of a project to build local hospitals in order to move healthcare closer to people in a major Swedish city, or stakeholders (healthcare managers and/or professionals, politicians) to the project. In the case during COVID‐19, 29 semi‐structured interviews were collected from healthcare managers and professionals across Sweden. Unlike the pre‐COVID‐19 interviews, these interviews were conducted through web‐camera to minimize the risk of infection and to avoid adding an extra burden on staff. In both cases, a qualitative approach was chosen because we were interested in subjective considerations and in understanding the phenomenon *healthcare closer to citizens* based on the respondents’ situation, position, and expertise (Alvesson, 2003; Thomsson, 2010).

**FIGURE 1:**
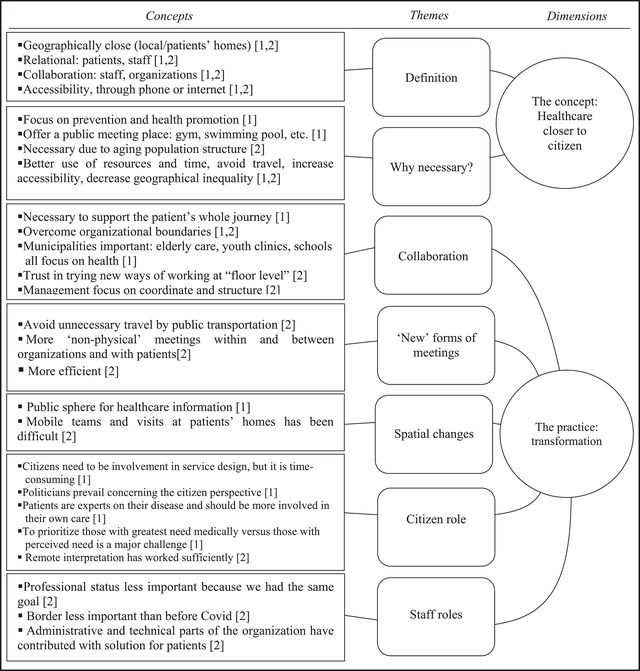
Coding structure

The interviews were transcribed verbatim and coded with inspiration from the so‐called Gioia methodology (Corley & Gioia, 2004; Gioia et al., 2013). First, transcribed interview data were analyzed and sorted based on similarities by staying as close to the respondents’ expressions and vocabulary as possible, and were sorted into *first‐order concepts*. Next, these concepts were compared and sorted into *second‐order themes*. Finally, overarching *aggregate dimensions* were developed. Figure 1 illustrates the constructed categories.

To ensure that nothing had been misunderstood, misinterpreted, or put in an incorrect context, the material was validated (Greenwood & Levin, 2007; Lincoln & Guba, 1985) during and after the coding process in presentations and discussions of initial findings with key stakeholders, some of whom had also participated in the interviews.

## 4 FINDINGS

In Figure 1, the number in brackets corresponds with the phase: 1 for pre‐COVID‐19 and 2 for during COVID‐19, enabling comparison between the two states.

### 4.1 Pre‐COVID‐19

As mentioned above, the context for this case was conducted in relation to the establishment of new local hospitals. To address the broader life situation, other than just treating the disease, such healthcare facilities should offer not only healthcare services, but also services such as a gym, café, and meeting places. While other, ‘non‐traditional’, actors’ services should be included in the hospital concept, some respondents argued that this was not a hospital's task: ‘As a hospital, you don't get any money for disease prevention’.

At the same time, the physical aspect of closeness of a local hospital was a limitation, building two local hospitals in a city with more a million inhabitants would, in reality, bring closeness only to a few citizens. Other respondents argued that physical closeness was not only related to where one lived, but just as importantly where one worked, spent time when off work, and so on. However, closeness could also be achieved by using information technology for communicating, which was underused today and would bring the patient closer to the staff. However, the relational aspect of closeness required a certain level of continuity in meeting staff physically.

An important aspect of closeness regarded citizens and patients. Some of the respondents involved in the project group expressed frustration that they had not been allowed to involve citizens in the design of the local hospitals because the politicians had made it clear that they wanted to do that themselves. There was a concern that genuine decision‐making would suffer and become a ‘paper product’, since politicians tended to focus on ‘numbers and figures’. The importance of listening in genuine dialogue was stressed by an official who had experience with citizens’ dialogues in order to get their perspective into the design of healthcare services. One politician admitted that the politicians had not involved citizens at all, instead being content to discuss the new hospital with public officials from different organisations. It was argued that, to a certain extent, the politicians could represent citizens on a broad level, but rarely patients who often had a different perspective. One interviewee argued that politicians insinuated ‘that citizen and patient involvement would be a barrier in the process’ and that there were expectations on the officials to be efficient.

Another aspect involved patient involvement in their own care and treatment, something that was argued would be more common due to technology as well as legislation enabling treatment at home, that patients took over some routine tasks, etc. – this required increased collaboration between actors, such as when treating patients in their homes.

Many of the respondents argued that it was not about building new local hospitals, but rather a healthcare *system*. This would challenge the dominant way of working for the healthcare actors as ‘isolated islands’. In such a system, it was important to provide functions and competences close to people, which required collaboration. Thus, overcoming organisational boundaries was a major challenge in creating such system, which had proved difficult in recent years because all actors had ‘different budgets, traditions and everything’. It was mentioned that the only way to achieve true collaboration was to merge all the budgets into one. Overall, there was a need for better coordinated care and improved collaboration in the actors in the decentralised system to increase coherency for the patient. Still, there were many unsolved problems that had been discussed ‘for years and years’, including different system for patient records between the actors, as well as difficulties in getting quick and easy access to other organisations to discuss issues related to a patient.

It was argued that the primary care provider, being a central actor, needed be provided with more resources, and primary care representants felt that they were not currently being compensated accurately and that the reimbursement system needed to be changed. Municipalities were sometimes neglected and had to be perceived as a natural actor in such healthcare system, not least because they were responsible for care of elderly at home or special accommodations and this population segment was growing, but also providing health information at school. The systems view was important because there was a risk that the system would become too large to handle, some respondents argued. Others argued that actors other than healthcare actors – such as the social insurance agency, public employment agency, and patient associations – should be involved in some way because they could provide important knowledge and information to understand the holistic perspective of the citizen's life situation. The presence of these actors at the hospital, maybe once a week, was essential for the holistic view of the system and the well‐being of patients. Creating touchpoints between actors was important for the local hospitals.

### 4.2 During COVID‐19

The understanding of ‘close healthcare’ was similar to the responses in the case before COVID‐19, including physical, relational, and organisational aspects. Whatever definition was used, many respondents argued that the importance of the concept of closer care had been revitalised during the pandemic. One manager argued that COVID‐19 had helped to make a diffuse and abstract strategy more concrete: ‘How can we make it operationalizable – what does it mean in practice?’ Trust was a key feature, not least in the national public health agency and regional infection control bodies, and local managers and staff followed their lead.

Several respondents felt that their own management function largely focused on ‘coordinating and structuring and at management level finding agreements with collaboration partners’. This may have been one reason why many perceived that boundaries – not only within organisations (units and professions), but also between organisations – had largely diminished in importance during this period. Several respondents felt that the leadership at different levels and in different parts (such as administration, IT support, and clinical work) had focused on the same issue and goals in the pandemic in a way that had not been done before. Some who worked close to patients had been involved in preparing documents for how the organisation would work during the COVID‐19 pandemic. Others similarly explained that they ‘worked closely with the managers’ during the pandemic: ‘The decisions were made in a sense of urgency for what we needed to achieve’.

Most of those who worked with patients felt that ‘there were no directives from the top of their own management’ or ‘… nothing came from politics or from the regional director or so’ and, at the same time, that no one from above interfered with ‘details’. The role of patient‐oriented leadership had been strengthened and the ‘floor level’ felt that it had a mandate to ‘solve the task on the spot’, without having to ‘wait for decisions from above’. The fact that no one ‘from above’ decided how things should be done was noted as a prerequisite for creating the commitment to develop new solutions themselves. Several respondents used the word ‘test’ was used by several respondents: ‘We have been allowed to test this in a way that we ourselves have had to think out, and I think that has been a success factor for us’.

The number of non‐physical meetings had increased substantially during the pandemic: with patients, within organisations, and between organisations. Digital media was mentioned much less than telephone, which was mainly described as important and said to save time compared to physical meetings, and also avoided the lack of clarity often found in e‐mails; one respondent said, ‘I do not think I have talked as much in phone in ten years!’ Non‐physical channels for communication had also worked exceptionally well for three‐party conversations, for instance with a foreign language or sign language interpreter present.

In line with the reform, visits to patients’ homes also increased during the pandemic, including visits to older patients. A positive side effect was the decreased risk of spreading the coronavirus by preventing patients and relatives from using public transportation or visiting hospitals. These visits often included staff from different levels of care, and it was evident that those who had well‐established relations prior to COVID‐19 could start new collaborations faster and more easily than those who did not.

As with organisational boundaries, boundaries between professions had diminished in importance during the pandemic. The importance of a pragmatic approach and new ways of working often meant a need to work together in a purely practical way, which reduced the difference between professional roles. Especially regarding the role of doctor, one could not hold on to the normal professional roles: ‘It's purely practical, in the wards where you have COVID patients … if the doctor puts on full protective equipment and goes into the room, the doctor also needs to bring the empty lunch tray back, for example’.

During the interviews, most respondents expressed a desire to maintain the new, or increased, practices developed during the pandemic: the increased levels of non‐physical meetings,^1^ provision of care at patients’ homes, and working across borders. Besides more focused meetings, the benefit also included less administrative burden and bureaucracy. In general, many judged the conditions for changing accepted working methods to be better now than before the pandemic and they experienced ‘a much better conversational climate now’. The non‐hierarchical and interprofessional practices that have been developed were also considered important to develop further in the transformation to healthcare closer to people.

## 5 DISCUSSION

The discussion has two foci. The first is the impact of COVID‐19 on speeding up the sought‐after transformation of the healthcare ecosystem to be close healthcare by enabling resource integration between professions and organisations. The second is to highlight the resources that are not being used in this transformation.

### 5.1 COVID‐19 as an ecosystem disruptor

The COVID‐19 situation in Sweden has accelerated much of the transition of the reform of moving healthcare closer to people. In doing so, central features of public service logic have also been highlighted, most notably collaborations across boundaries (Eriksson & Hellström, 2021). The case prior to COVID‐19 clearly shows how formal and informal rules and regulations (Akaka et al., 2013; Vargo & Akaka, 2012) hindered collaborations regarding moving healthcare closer to people. The role casting between politicians and officials entailed that the latter could not include citizens in developing services, reimbursement systems made it difficult for primary care to collaborate, hospitals were not paid to prevent disease, and so forth. It was mentioned in the interviews that this type of collaborations had been sought after, unsuccessfully, for years.

To a large extent, it has been argued that the sense of urgency due to the COVID‐19 pandemic has improved collaborations, not least building on good relationship and trust (e.g., Agranoff & McGuire, 2004; Provan & Kenis, 2008). Thus, coordinating services by integrating resources – between healthcare providers at different levels in the system, as well as between professions – through collaborations was important to avoid patients falling through the cracks (Eriksson et al., 2020), something prioritised by managers being busy structuring and coordinating, enabling collaboration at the micro‐level by trusting their employees and giving them a mandate to ‘test’ developing practices themselves and sometimes work closely with management to develop guidelines and so forth. The ideal of healthcare professionals expressed in the quality improvement literature showed empirically that healthcare staff have two jobs: ‘to do their work and to improve it’ (Bataladen & Davidoff, 2007, p. 3).

During the pandemic, the healthcare professionals’ power or latitude may have increased in relation to the predominant management discourse (Doolin, 2002; Malmmose, 2015). Moreover, teamwork between professionals improved as boundaries between professionals (Mintzberg, 2017) became more diffuse.

Also interesting is what was mentioned before the pandemic, but not at all during the pandemic. For example, reimbursements systems (Mandell & Keast, 2008), data systems for records, and competition (Grönroos, 2019) as aspects hindering collaboration were common before COVID‐19 and in the literature, but absent in the interviews during the pandemic. Formal rules and different budgets have also been commonly identified as obstacles (Eriksson et al., 2020), but absent in the empirical material during the pandemic.

COVID‐19 has revealed some weaknesses in the Swedish healthcare ecosystem: decentralisation of elderly care and its lack of medical competence, regulations, and accountability between healthcare providers, similar to the findings in the Swedish Corona Commission (2020a). Moreover, unexpected aspects of management fashions (Alvesson & Spicer, 2012) such as the just‐in‐time stocking, and inequities in that certain segments of the population had a greater risk of contracting COVID‐19. To a certain extent, however, the ecosystem proved to be ‘adaptable, agile and pragmatic’ (Ansell et al., 2020, p. 1). However, probably because of the sense of urgency, the respondents were more solution‐orientated than reflecting.

### 5.2 A delimited ecosystem

Rather than organisational matters, a central feature in public service logic is what happens for the service user (Osborne, 2020). Fewer respondents during the pandemic emphasised the citizens’/patients’ perspectives than in the interviews before the pandemic. There is no increase in patient involvement in any phase of the policy cycle in the amidst COVID‐19 case compared to before. Indeed, the traditional role casting has been cemented due to COVID‐19, healthcare provides services and the citizens are not involved in developing facilities and services (Jacquez et al., 2013; Olsson et al., 2014), which may be natural in a sense of urgency situation. The interviews during COVID‐19 contained little discussions about the patient's home situation. In addition, public spheres as areas for healthcare provision and consultation were not mentioned in the interviews. The respondents mentioned that COVID‐19‐related information was mainly provided through authorities’ websites, which may have led to certain groups (such as older patients and foreign‐born inhabitants) missing information more than others.

Overall, it was mentioned that few new services were created during the pandemic; instead, established practices (mobile teams visiting patients at their homes, telephones, interpretation on distance) were used more frequently than before. It is noteworthy that mainly traditional telephone calls were used rather than digital communication applications, even though digital services were mentioned as important for people to access public services (Dunleavy et al., 2006; Melchiorre, 2018) as well as in the interviews before the pandemic. Leite and Hodgkinson (2021) found that telemedicine, or face‐to‐face video consultations, may be a way for patients and healthcare staff to co‐create the service in a public service fashion during the pandemic. Moreover, the inclusion of the citizens’ and patients’ perspectives would have led to new and innovative ways of carrying out healthcare services during pandemic (Eriksson, 2019).

More non‐physical visits per telephone were perceived as positive and few reflected on that statement. Only in a few cases was it mentioned that physical and non‐physical meetings may serve different purposes and the need for either type of meeting varies among patients and patient groups. It has been argued that while non‐physical meetings may be efficient (as in saving time in the short run), relationship building in the long run may suffer (Morgan & Yoder, 2012), and this is an essential aspect of both public service logic (Osborne, 2020) as well as healthcare‐specific approaches such as person‐centered care (Ekman et al., 2011). Moreover, loneliness has been highlighted as a major challenge during the pandemic, not least for the elderly. In addition, it was mentioned in the interviews during the pandemic that the new technologies could risk excluding older patients further.

Just like blurred boundaries, the benefits of less bureaucracy (in terms of specific rules and regulations) is mentioned as a unilaterally positive consequence of the pandemic. However, the lighter administrative burden experienced by staff (Moynihan et al., 2015) may be unintentionally accompanied by decreased patient safety, data security, and inequality when protocol may risk being replaced by an arbitrary and *laissez faire* culture – which an important task of bureaucracy is to counteract (e.g., Du Gay, 2000).

Through depoliticisation (e.g., Clarke, 2013) it is often argued that management has gained power at the expense of politicians (Mattei, 2006). Similarly, the political dimension is not mentioned in the interviews during COVID‐19, but clearly problematised before the pandemic.

Moreover, the healthcare ecosystem may actually shrink, in regards of involved actors, in sense of urgency. In the interviews during the pandemic, the respondents mentioned fewer actors in the ecosystem than they did before the pandemic. For instance, school and youth clinics were not mentioned at all during the pandemic.

## 6 CONCLUSION

The present paper has contributed to the development of the public service logic by recognizing how turbulent times, such as the COVID‐19 pandemic, may accelerate reforms in a desired direction, even reforms that have been stationary for quite some time. However, the paper has also highlighted that expected enablers for value co‐creation in these service ecosystems, such as telemedicine (Leite and Hodgkinson (2021), may be underused, despite the sense of urgency. The findings are also transferable to other overlapping governance concepts. The practical contribution is that the Swedish atypical case can inform a global audience not only about healthcare reform in a COVID‐19 context but also during other turbulent times.

Having studied the transformation of the healthcare system towards closer healthcare before and after the pandemic, it is clear that COVID‐19 has functioned as a ‘game changer’ (Ansell et al., 2020) or ‘disruptor’ (Christensen et al., 2018) of Swedish healthcare by challenging predominant ways of doing things. As seen, clearly desirable aspects of closer healthcare are happening due to the COVID‐19: boundaries within and between organisations are becoming less important, which is increasing collaborations based on trust and relationship; staff are given mandates to try new ways of working; and so forth. Paradoxically, the sense of urgency also seems to have created an inwardness among the established organisations within the healthcare ecosystem – healthcare providers – at the expense of the impact by citizens/patients and other service providers, which are all central in the narrative before the pandemic. Maybe more new services would be identified if patient/citizens had been more involved in developing pandemic‐specific practices from their situation and life situation.

Several of the interviews emphasised the importance of a solution focus and a positive spirit as a necessity for dealing with the emergency situation. This was probably a reason for the lack of reflection in the interview material. For example, a relatively high amount of argumentation was done in terms of either–or, rather than it‐depends‐on (telephone visits may not always be able to replace physical visits, lack of rules and boundaries may obscure responsibilities and allow arbitrariness, etc.).

As was the case for the respondents, it can be challenging to study a phenomenon just when it happens. Therefore, future research could explore experiences after the pandemic as the respondents have been able to take a step back and reflect on approaches and working methods. Reflecting as things happen, and at a time when normal assessment criteria are put out of play, is admittedly difficult (Schön, 1983) – and should apply especially in this atypical situation. For policymakers and practitioners, this paper has highlighted organisation and management during these extraordinary and ‘turbulent’ circumstances, as well as the need to thoroughly consider certain actions.

## CONFLICT OF INTEREST

The author(s) declared no potential conflicts of interest with respect to the research, authorship, and/or publication of this article.

## Footnotes

1 During the presentations of the initial results, when the situation had settled somewhat, it was mentioned that it was now mainly managers who wanted to keep the high number of non‐physical meetings, with frontline staff greatly wanting to go back to how things were before COVID‐19, mainly in terms of meeting patients physically.
